# Targeted RNA sequencing identified gene expression profiles linked to severe necrosis and mortality in preterm infants with surgical necrotizing enterocolitis

**DOI:** 10.21203/rs.3.rs-7244063/v1

**Published:** 2025-07-31

**Authors:** Parvesh Mohan Garg, David Sawaya, Robin Riddick, Seth Lirette, Nicole Hall, Neha Varshney, Timothy D. Howard, William B Hillegass, Akhil Maheshwari, Padma Garg

**Affiliations:** Department of Pediatrics/Neonatology, Wake Forest University School of Medicine, Winston Salem, North Carolina; Department of Pediatric Surgery, University of Mississippi Medical Center, Jackson, Mississippi; Department of Pediatrics, University of Mississippi Medical Center, Jackson, Mississippi; Department of Biostatistics, University of Mississippi Medical Center, Jackson, Mississippi; Department of Biochemistry, Wake Forest University School of Medicine, Winston Salem, North Carolina; Department of Pathology, University of Mississippi Medical Center, Jackson, Mississippi; Department of Biochemistry, Wake Forest University School of Medicine, Winston Salem, North Carolina; Department of Biostatistics, University of Mississippi Medical Center, Jackson, Mississippi; Neonatology, Boston Children’s Physicians Group at New York Medical College and Maria Ferrari Children’s Hospital, Westchester, NY; Department of Pediatrics, University of Mississippi Medical Center, Jackson, Mississippi

**Keywords:** Preterm Infants, NEC, Necrosis, Mortality, Gene Expression, RNA sequencing, Neonate

## Abstract

**Background:**

We aim to determine the gene expression changes that occur in surgical NEC infants with and without moderate to severe necrosis and survivors and non-survivors.

**Methods:**

Targeted RNA sequencing was performed on RNA isolated from formalin-fixed, paraffin-embedded (FFPE) intestinal tissue samples (N=36). DeSeq2 was used to analyze differential expressions between infants with mild to moderate and severe necrosis and with respect to survival status.

**Results:**

Thirty-five genes were differentially expressed (FDR- adjusted p < 0.05) between mild-medium necrosis and severe necrosis. Genes involved in altered host defense, natural killer (NK) cell signaling and development, and apoptosis were overexpressed in severe necrosis (*IGJ, GZMA, TNFSF10, KLRB1*, and *CD160*). Expression of leukocytes antigens (*ITGAM, ITGAX*) and cytokine and chemokine receptors (such as *IL1A, IL1B, CCL2, CCL3*) were increased in patients with mild necrosis. Six genes were significantly differentially expressed (FDR- adjusted p < 0.05) between survivors and the non-survivors. Genes related to chemokines attracting neutrophils (*CXCL1, GBP,PTGS2,CXCL11,CXCL9*, and *CXCL10*) were upregulated in non-survivors.

**Conclusion:**

Severe necrosis and non-survival of NEC infants were associated with differential genes expression related to host defense, NK cell signaling and development, and apoptosis. Understanding these pathways can guide the development of prognostic and treatment pathways.

## Introduction

Necrotizing enterocolitis (NEC) is a devastating acute gastrointestinal illness of the neonatal period, affecting about 5–10% of premature infants with birth weights of ≤1500 grams^1,2^. Despite major advances in neonatal intensive care and reductions in all-cause mortality among premature infants, NEC continues to be associated with mortality rates of 25–40%^3–8^.

Histopathologically, NEC lesions are marked by coagulative necrosis, inflammation, bacterial overgrowth, and reparative tissue changes^9–16^. A recent study correlated outcomes in NEC with the depth of bacterial invasion and the severity of inflammation in the resected bowel. In addition, the presence of NEC lesions in the resection margins, presumably indicating incomplete removal of necrotic bowel, was also associated with mortality^17^. There is also evidence that including genetic information may be helpful in predicting the disease outcomes in NEC. Twin studies suggest that genetic factors may account for up to 50% of the risk of NEC^18^. Certain single nucleotide polymorphisms SNPs, such as those in carbamyl phosphate synthetase (*T1405N*)^19^, *IL12* (p40 promoter CTCTAA/GC)^20^, *VEGF* (C-2578A)^21^, and *NFKB1*(g.−24519delATTG),^22^ have been found to be associated with NEC, but genome-wide association studies for NEC are very few^23^.

The challenge for neonatologists is to detect early clinical manifestations of NEC. One strategy would be to identify specific markers that could be used as early diagnostic tools to identify preterm infants most at risk of developing NEC or in the event of a diagnostic dilemma of suspected disease. As a step in this direction, we aimed to determine the immune gene expression changes associated with different severities of necrosis, in preterm infants with surgical necrotizing enterocolitis. Additionally, we aimed to determine the differential immune gene expression that occur in survivors and non-survivors in preterm infants with surgical NEC.

## Methods

This study was conducted at the University of Mississippi Medical Center (UMMC), under the oversight of the Institutional Review Board (protocol 2017–0127). The neonatal intensive care unit at UMMC is a regional referral center for infants with surgical NEC. We reviewed the medical and pathology records to identify patients with advanced NEC^24^ who underwent an exploratory laparotomy and surgical resection of the bowel during 15 years (January 2000 to December 2015). The patients with spontaneous intestinal perforation were excluded.

Demographic characteristics, including birth weight, gestational age, gender, race, mode of delivery, and out born status were retrieved from the patients’ medical records. Maternal data, including pregnancy-induced hypertension, chorioamnionitis, and antenatal steroid use was recorded. Infant characteristics, including mode of delivery, small for gestational age status, Apgar score at 5 minutes, age of NEC onset, mode of clinical presentation (bloody stools, abdominal distension, feeding intolerance), persistent patency of the *ductus arteriosus* (PDA), use of non-steroidal agents for medical treatment of PDA, assisted ventilation, blood culture-positive sepsis, and duration of antibiotics were recorded. The region of the affected bowel and length of bowel resected was also noted. We also collected data on the length of hospital stay and mortality. The length of hospital stay was defined as the total length of stay from the day of admission until discharge. Mortality was defined as death due to NEC or NEC-associated sepsis. The length of stay was defined as the total length of stay from the day of admission until discharge. To assess postoperative morbidity, we recorded the duration of postoperative ileus, days of parenteral nutrition days, development of short bowel syndrome (SGS), and time to achieve full feeds. Short bowel syndrome was defined as infants who were still requiring TPN more than 90 days following the NEC surgery. Days of parenteral nutrition were defined as the interval between postoperative day 0 until full enteral feedings were achieved (defined as 120 ml/kg/day). Surgical morbidity was classified as surgical site infections (including dehiscence and abscesses), strictures, fistulas, adhesions, and perforations.

### Histopathological Evaluation:

Specimens of surgically resected bowel tissue from the patients with NEC were identified in pathology archives. Hematoxylin and eosin-stained resected bowel tissue sections were recorded for the area of necrosis on the slide (percent), scored as 0, 1 (0–25%), 2 (26–50%), 3 (51–75%), and 4 (76–100%); maximum depth of necrosis, scored as 0, 1 (mucosa), 2 (submucosa), 3 (*muscularis propria*), and 4 (transmural); severity of inflammation, scored as 0 (no inflammatory cells), 1 [up to 50 inflammatory cells/high power field (hpf)], 2 (51–200 inflammatory cells/hpf, 3 (>201 cells/hpf); depth of inflammatory infiltrates, scored as 0, 1 (mucosa), 2 (submucosa), 3 (muscularis propria), and 4 (transmural)^17^.

### RNA Sequencing Methods:

RNA was isolated from 10um section of NEC formalin-fixed, paraffin-embedded (FFPE) human intestine specimens (N=36) obtained following laparotomy Targeted RNA sequencing using the AmpliSeq Illumina Immune Response Panel was utilized for targeted quantitative expression of genes linked to immune system interactions which included 400 genes. Raw reads were de-multiplexed and aligned to human reference genome assembly (hg19) using RNA Amplicon Application (along with custom panel manifest) available on Illumina BaseSpace Computing Platform (DRAGEN APP; http://basespace.illumina.com/). Aligned reads for each gene were normalized for sequencing depth and RNA composition using DESeq2’s median of ratios method and used for downstream differential expression analysis in survivors and the non -survivors while accounting for other covariates such as necrosis.

### Statistical Analysis:

Descriptive statistics were computed; categorical data was summarized as frequencies (absolute and relative) and any simple bivariate comparisons were performed using Fisher’s exact tests or chi square tests. Continuous data were presented as either means with standard deviations, when underlying distributions would be roughly symmetric, or medians with first and third quartiles when skew was noted. Differences in the continuous data were tested using a student’s t-test or if symmetry was in question, a Mann-Whitney *U* test. A p-value of less than 0.05 was considered significant.

## Results

Clinical data of the 36 infants included in the study are summarized in Table 1. The cohort had mean birth weight of 1005 gm (SD 555 gm) and the mean gestational age of 27.2 weeks (SD3.0). 23 infants were males, and 13 infants were female in the study cohort. In the study 21 infants (21/36, 58.3%) had mild to moderate necrosis and 15 infants (15/36,41.6%) had severe necrosis (score 3–4). Out of 36 infants with surgical NEC, 11 infants (11/36, 30.5%) had died, and 25 infants (25/36, 69.4%) had survived and were included in the analysis. **The data are summarized in** Table 1.

### Gene Expression in infants with mild-moderate necrosis and severe necrosis:

#### RNA-Seq analysis and identification of differentially expressed genes (DEGs):

**Figure 1:**
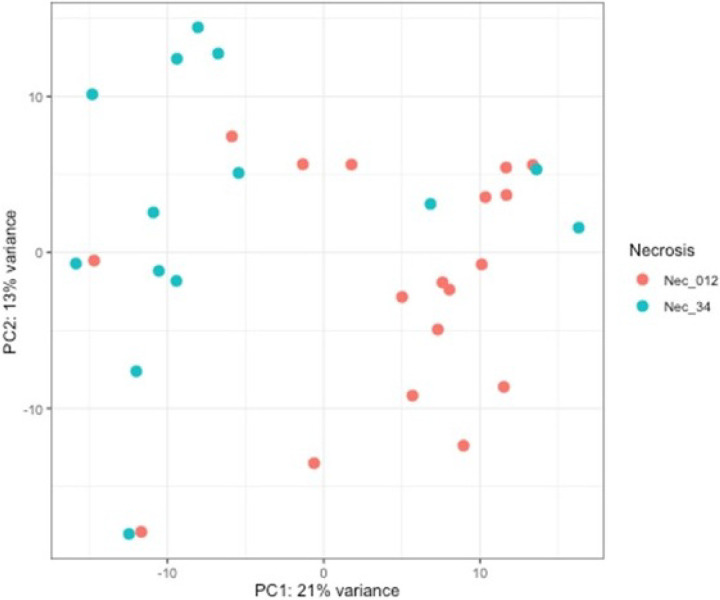
Principal component analysis. PCA analysis showing gene expression variability in PC1 and PC2 with low necrosis (orange) and high necrosis (blue) infants.

**Figure 2:**
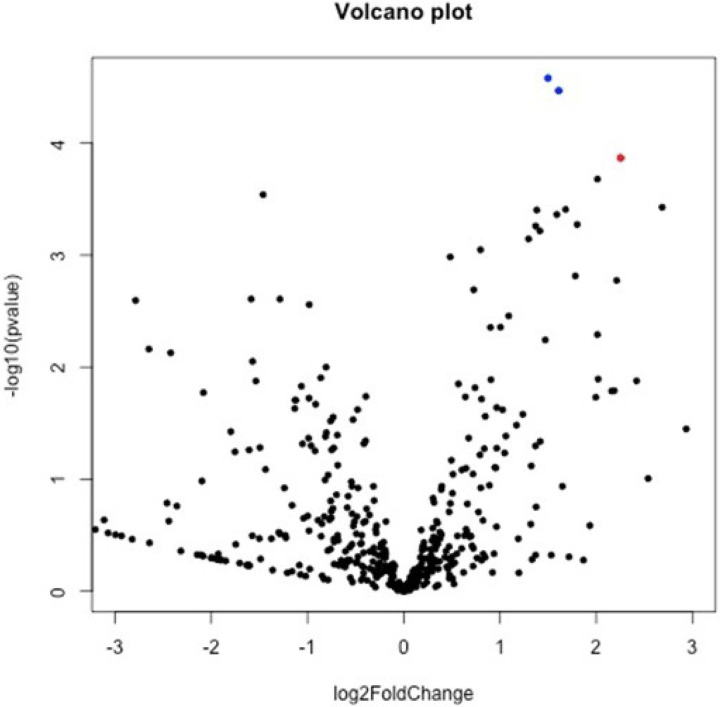
Volcano plot analysis for necrosis DEGs. The *X* axis represents the log2 transformed difference fold value, and the *Y* axis represents the −log10 transformed significance value.

**Figure 3:**
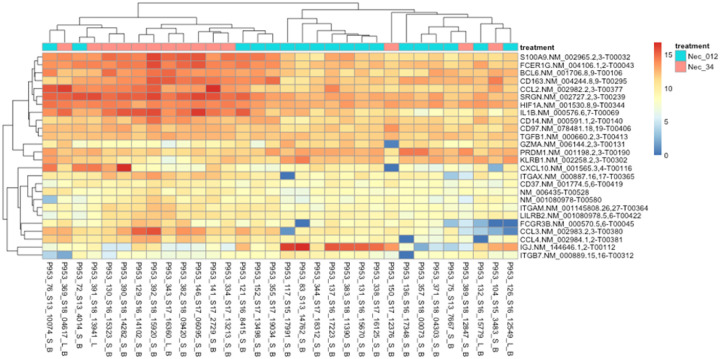
Heat Map of necrosis DEGs. Rows represent the differential gene expression of the listed gene, and columns represent the individual patients. Necrosis levels are indicated by blue (low) or red (high) colors.

Differential expression analysis was used to investigate the association with stage of necrosis and gene expression in preterm infants with NEC. The analysis showed 35 genes were differentially expressed (p < 0.05) between mild-medium necrosis (score 0–2) and severe necrosis (score of 3–4). A majority of the amplicons included in the panel were detected. Genes related to altered host defense, natural killer cell signaling and development, apoptosis etc. were overexpressed in severe necrosis (*IGJ, GZMA, TNFSF10, KLRB1, CD160*). Expression of leukocyte antigens (*ITGAM, ITGAX*) and cytokines and chemokines receptors (*IL1A, IL1B, CCL2, CCL3*) were upregulated in patients with mild to moderate necrosis. **The data are summarized in** figure 1–3.

### Gene expression in survivor and non-survivor:

#### RNA-Seq analysis and identification of DEGs:

Differential expression analysis showed six genes being significantly differentially expressed (FDR adjusted p < 0.05) between survivors and the non-survivors. Genes related to chemokines attracting neutrophils (*CXCL1*), *GBP1* (Guanylate binding protein −1, role in cell-autonomous immunity) and *PTGS2* (prostaglandin-endoperoxide synthase 2 gene, encodes for the cyclooxygenase-2 (*COX2*) enzyme, needed for prostanoid biosynthesis involved in inflammation and mitogenesis), *CXCL11* (cellular response to lipopolysaccharide; chemokine-mediated signaling pathway; and neutrophil chemotaxis), *CXCL9* (Leukocyte chemotaxis, recruitment and differentiation), and *CXCL10* (recruits immune cells like macrophages, DCs, NK cells, and Th1 cells to sites of inflammation) were all increased in non-survivors.

**Figure 4:**
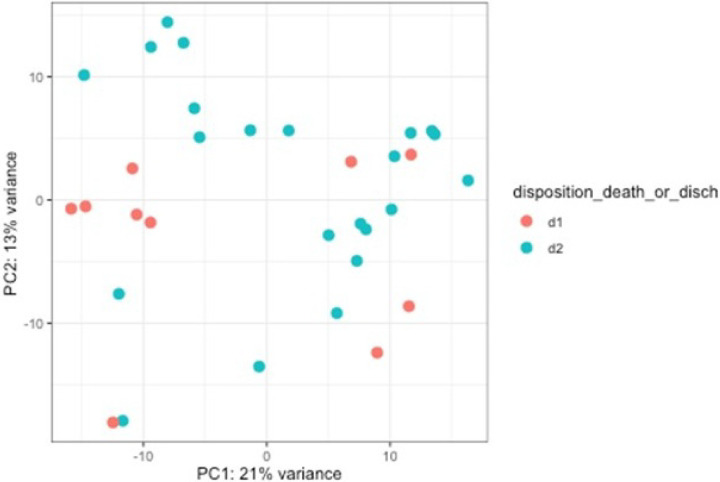
Principal component analysis: PCA analysis shows gene expression variability in PC1 and PC2 with respect to survival status.

**Figure 5:**
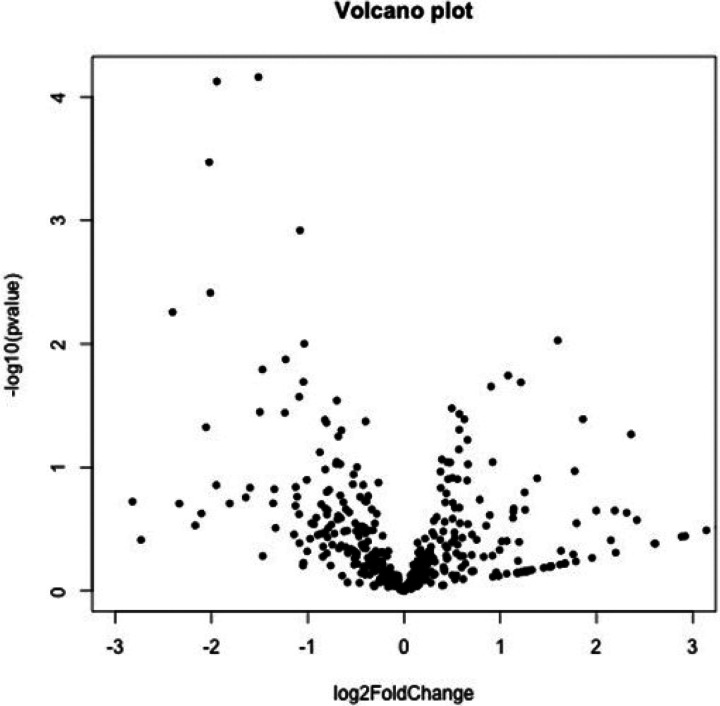
Volcano plot analysis with respect to survival and non- survival status DEGs. The *X* axis represents the log2 transformed difference fold value, and the *Y* axis represents the −log10 transformed significance value.

**Figure 6:**
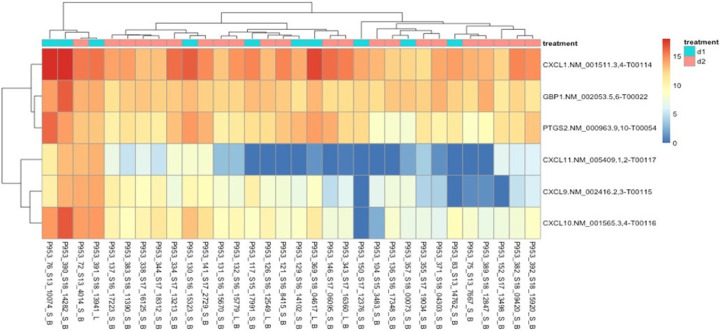
Heat Map of DEGs in relation to survival status. Rows represent the differential gene expression of the listed gene, and columns represent the individual patients.

**Figure 7:**
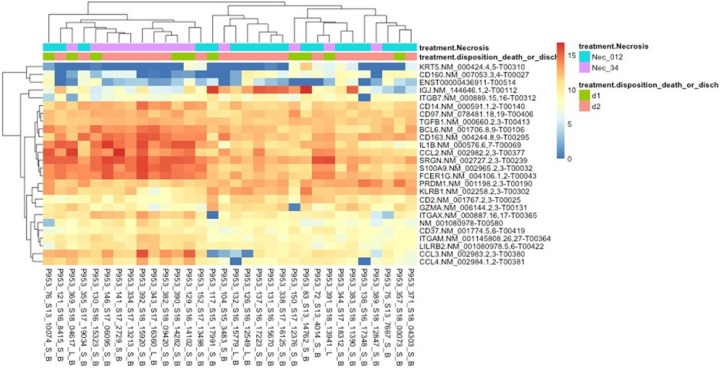
Heat Map of DEGs in relation to necrosis and survival status. Rows represent the differential gene expression of the listed gene, and columns represent the individual patients.

When tested and accounted for the severity of necrosis contributing to the outcome, 26 genes were differentially expressed between survivors and non-survivors. Genes involved in epithelial cell structure, altered host defense and immune cell signaling and development (*IGJ, GZMA, KLRB1, KRT5* and *CD160*), were all downregulated in patients with severe necrosis, suggesting a suppressed immune response compared to those with less severe necrosis. On the other hand, expressions of leukocyte antigens (*ITGAM* and *ITGAX*) and cytokines and chemokines receptors (*IL1B, CCL2, CCL3*, and *CCL4*) were upregulated in patients with mild to moderate necrosis. **The data are summarized in** figure 4–7.

## Discussion

Our study investigated the gene expression underlying the severe necrosis and death in preterm infants with surgical NEC, by utilizing mRNA sequencing from formalin-fixed paraffin-embedded intestinal tissue samples. Our results showed that severe necrosis (based on intestinal pathology) in NEC infants was associated with increased expression of genes related to host defense, natural killer cell signaling and development, and apoptosis. Our data also shows that survivors in surgical NEC patients showed distinct immune gene expression profiles, with non-survivors exhibiting increased inflammation and chemotactic signaling. Survivors had increased gene expression of leukocyte antigens and cytokine receptors, indicating a more active immune response relative to non-survivors. The degree of necrosis was associated with additional genes, emphasizing its role in immune regulation differences.

Tremblay et al^25^ analyzed RNA expression in ileal samples from preterm infants with NEC and compared them to samples from infants with other diseases. They found that NEC intestines showed over-representation of pathways related to innate immunity, such as altered T and B cell signaling, granulocyte adhesion and diapedesis, B cell development, and pattern recognition receptor roles. ToppCluster analysis further revealed that NEC was marked by increased lymphocyte and leukocyte migration, chemotaxis, and adhesion, while functions related to lipid metabolism were down-regulated, defining a distinct biological signature for NEC^25^. We also identified genes related to altered host defense, natural killer cell signaling and development, and apoptosis as being overexpressed in surgical NEC infants with severe necrosis, relative to the others.

Tremblay et al^25^ reported the up-regulation of *CXCL10, TLR4, TLR10, DEFA5, REG3A, LCN2* and *TFF3* and down-regulation of *HBA2* and *HBG2* expression in NEC. They also reported a high degree of similarity between NEC and Crohn’s disease gene expression^25^. Similarly, in our cohort, *CXCL11* (cellular response to lipopolysaccharide; chemokine-mediated signaling pathway; and neutrophil chemotaxis), *CXCL9* (leukocyte chemotaxis, recruitment and differentiation), and *CXCL10* (recruit’s immune cells like macrophages, DCs, NK cells, and Th1 cells to sites of inflammation) were all increased in non-survivors.

A recent study by Xie et al. analyzed RNA-Seq data from intestinal tissues of 12 preterm infants with NEC, NEC self-control, and normal controls, detecting 34,712 genes and identifying 7,463 differentially expressed genes (DEGs). Gene Ontology analysis showed these DEGs were mainly involved in chemokine receptor binding, transporter activity, and growth factor binding, while KEGG analysis indicated significant enrichment in toll-like receptor signaling, Th17 cell differentiation, and cytokine–cytokine receptor interaction pathways. Immune infiltration profiles differed notably among the three tissue groups^26^.

A recent study analyzed 129 formula-fed preterm piglets diagnosed with necrotizing enterocolitis (NEC) at necropsy on day 5, comparing subgroups of NEC (*n* = 20) and control piglets (*n* = 19) via whole-blood transcriptome analysis^27^. Lesions were scored on a 1–6 scale, with scores ≥3 indicating NEC. Severe cases (scores 5–6) showed transmural necrosis or pneumatosis intestinalis^27^. 344 differentially expressed genes (DEGs) were identified between NEC and control groups, highlighting systemic immune responses and inflammation. Validation confirmed AOAH, ARG2, FKBP5, PAK2, and STAT3 as genes significantly altered in severe NEC cases, detectable in both whole blood and dried blood spots (DBS)^27^. In summary, blood gene expression changes occurred before severe clinical manifestations, suggesting potential for early NEC detection. DBS sampling emerged as a feasible method for small-volume blood collection in infants, enabling practical biomarker screening^27^.

The strength of our study is that we have provided a unique perspective, as we compared gene expression in preterm infants with surgical NEC with varying degrees of disease severity, whereas other studies have compared NEC patients to healthy controls. Some limitations of our study are that our samples were collected from a single center, our sample size was small, and we did not include lack of healthy controls. In addition, FFPE samples typically have poorer quality RNA, which may have biased our results towards more stable transcripts. Finally, due to the design of the study we are unable to demonstrate a cause-and-effect relationship, so it is unclear if the expression differences observed between groups are due to the primary phenotype or a systemic effect due to the disease process.

## Conclusion

In conclusion, this study has led to the identification of several DEGs in intestinal samples of premature infants affected with NEC that could be of clinical interest as potential biomarkers for the prediction of the disease and its diagnosis.

Survivors and non-survivors with surgical NEC show distinct immune gene expression profiles, with non-survivors exhibiting increased inflammation and chemotactic signaling. Survivors have upregulation of leukocyte antigens and cytokine receptors, indicating a more active immune response. Degree of necrosis impacts additional genes, emphasizing its role in immune regulation differences. Targeting excessive inflammation and understanding these pathogenic pathways may improve outcomes.

## Figures and Tables

**Table 1 :** Clinical information of surgical NEC preterm infants with and without severe necrosis and in relation to survivors and mortality

	Total n=36	Death n=11	Discharge n=25	p	Necrosis 0–2 n=21	Necrosis 3–4 n=15	p
**Appropriate Gestational Age, n (%)**	36	8 (72.7%)	10 (40%)	0.07	11 (52.4%)	7 (46.7%)	0.5
**Gestational Age (weeks, mean (SD))**	36	27.2 (2.98)	27.3 (3.07)	0.92	26.4 (3.11)	28.5 (2.47)	**0.043**
**Gender, n (%)**	36			0.63	9 (42.9%)	4 (26.7%)	0.26
**Male**		7 (63.6%)	16 (64%)		12 (57.1%)	11 (73.3%)	
**Female**		4 (36.4%)	9 (36%)		9 (42.9%)	4 (26.7%)	
**Ethnicity, n (%)**	36						0.37
**Caucasian**					5 (23.8%)	5 (33.3%)	
**African American**					14 (66.7%)	9 (60.0%)	
**Hispanic**					0 (0.0%)	1 (6.7%)	
**Other**					2 (9.5%)	0 (0.0%)	
**Mode of Delivery, n (%)**	36			0.25			0.52
**C-section**		9 (81.8%)	16 (64%)		15 (71.4%)	10 (66.7%)	
**Vaginal**		2 (18.2%)	9 (36%)		6 (28.6%)	5 (33.3%)	
**Birth Weight (g, mean (SD))**	36	1005.2 (655.05)	1006.3 (520.63)	0.99	900.8 (570.82)	1153.3 (515.60)	0.18
**Outborn, n (%)**	36	5 (45.5%)	16 (64.0%)	0.25	12 (57.1%)	9 (60.0%)	0.57
**Length of Stay (days, mean (SD))**	36	93.0 (94.24)	146.0 (60.87)	**0.022**	145.9 (60.86)	123.9 (96.27)	0.41
**Time to surgery from NEC onset, (hrs, mean (SD))**					355.7 (401.45)	88.6 (99.33)	**0.017**
**Clinical Presentation of NEC, n (%)**	36			0.1			0.08
**Abdominal Distention**		8 (72.7%)	24 (96%)		20 (95.2%)	12 (80.0%)	
**Bloody Stools**		2 (18.2%)	1 (4.0%)		0 (0.0%)	3 (20.0%)	
**Feeding Intolerance**		1 (9.1%)	0 (0.0%)		1 (4.8%)	0 (0.0%)	
**Portal Venous Gas, n (%)**	36	0 (0.0%)	2 (8.0%)	0.48	2 (9.5%)	0 (0.0%)	0.33
**Pneumatosis, n (%)**	36	7 (63.6%)	8 (32.0%)	0.08	6 (28.6%)	9 (60.0%)	0.06
**Pneumoperitoneum, n (%)**	36	4 (36.4%)	14 (56.0%)	0.24	10 (47.6%)	8 (53.3%)	0.5
**Length and Region of Bowel Resected (cm, mean (SD))**	36	22.7 (12.51)	27.6 (22.21)	0.5	18.5 (14.30)	36.8 (21.66)	**0.004**
**Region of Bowel Resected, n (%)**	36			0.26			
**Small Bowel**		7 (63.6%)	20 (80.0%)				
**Large Bowel**		1 (9.1%)	0 (0.0%)				
**Both**		3 (27.3%)	5 (20.0%)				
**Time to reach full feeds (mean (SD))**	25	67.0 (55.15)	81.5 (46.85)	0.68	83.6 (43.86)	74.4 (52.89)	0.65
**Central line (days, mean (SD))**					75.5 (45.94)	50.6 (36.83)	0.12
**TPN (days, mean (SD))**					110.7 (53.57)	78.7 (61.06)	0.11
**Pregnancy Induced Hypertention, n (%)**	36	4 (36.4%)	6 (24.0%)	0.35	6 (28.6%)	4 (26.7%)	0.6
**Chronic Hypertension, n (%)**	30	4 (50.0%)	3 (13.6%)	0.06	3 (15.8%)	4 (36.4%)	0.2
**Chorioamnionitis, n (%)**	36	11 (100%)	25 (100%)		0 (0.0%)	0 (0.0%)	
**Cholestasis, n (%)**	30				12 (70.6%)	7 (53.8%)	0.29
**Antenatal Steroids, n (%)**	36	8 (72.7%)	18 (78.3%)	0.52			
**Patent Ductus Arteriosus, n (%)**	36	7 (63.6%)	13 (52%)	0.39	13 (61.9%)	7 (46.7%)	0.29
**24 hour lonotropic support, n (%)**	36	9 (81.8%)	16 (64.0%)	0.25	14 (66.7%)	11 (73.3%)	0.48
**Platelets after NEC (mean (SD))**	35	100 (52.67)	152.4 (91.75)	0.09			
**Positive Blood Culture Sepsis, n (%)**	36	4 (36.4%)	9 (36%)	0.63	9 (42.9%)	4 (26.7%)	0.26
**Indomethacin Use, n (%)**	36	1 (9.1%)	3 (12.0%)	0.64			
**Surgical Complication, n (%)**	36	7 (63.6%)	10 (40%)	0.17	11 (52.4%)	6 (40.0%)	0.35
**Single Complication, n (%)**	36	3 (27.3%)	6 (24.0%)	0.57	6 (28.6%)	3 (20.0%)	0.43
**More than 1 Complication, n (%)**	36	2 (18.2%)	4 (16.0%)	0.61	4 (19.0%)	2 (13.3%)	0.51
**White Matter Injury, n (%)**	23	1 (33.3%)	9 (45.0%)	0.6	6 (42.9%)	4 (44.4%)	0.64
**Inflammation, n (%)**	36			0.81			0.2
**0%**		0 (0.0%)	3 (12.0%)		0 (0.0%)	3 (20.0%)	
**25%**		2 (18.2%)	5 (20.0%)		5 (23.8%)	2 (13.3%)	
**25–50%**		6 (54.5%)	12 (48.0%)		12 (57.1%)	6 (40.0%)	
**50–75%**		2 (18.2%)	3 (12.0%)		3 (14.3%)	2 (13.3%)	
**>75%**		1 (9.1%)	2 (8.0%)		1 (4.8%)	2 (13.3%)	
**Hemorrhage, n (%)**	36			0.89			0.35
**0%**		0 (0.0%)	2 (8.0%)		0 (0.0%)	2 (13.3%)	
**25%**		1 (9.1%)	3 (12.0%)		3 (14.3%)	1 (6.7%)	
**25–50%**		5 (45.5%)	9 (36.0%)		7 (33.3%)	7 (46.7%)	
**50–75%**		3 (27.3%)	6 (24.0%)		6 (28.6%)	3 (20.0%)	
**>75%**		2 (18.2%)	5 (20.0%)		5 (23.8%)	2 (13.3%)	
**Heal, n (%)**	36	6 (54.5%)	11 (44.0%)	0.41	11 (52.4%)	6 (40.0%)	0.35
**Penrose Drain, n (%)**	35	4 (40.0%)	9 (36.0%)	0.56	10 (50.0%)	3 (20.0%)	0.07
**CRP on day of NEC (mean (SD))**	29	8.3 (10.87)	7.8 (10.19)	0.92	6.1 (5.64)	10.2 (13.88)	0.3
**CRP 24 hrs after NEC (mean (SD))**	25	15.1 (12.95)	12.3 (14.57)	0.68	7.6 (6.78)	19.8 (17.83)	**0.027**
**Disposition, n (%)**	36						0.25
**Death (due to NEC)**					5 (23.8%)	6 (40.0%)	
**Discharged**					16 (76.2%)	9 (60.0%)	

## Impact

1. The gene expression profiles in surgical necrotizing enterocolitis (NEC) were found to differ depending on necrosis severity and survival outcome.
2. Significant differences in gene expression levels helped to identify molecular pathways that may be regulated and involved in disease progression.
3. The differentially expressed genes and molecular pathways are potential therapeutic targets for NEC, an incompletely understood and life-threatening disease.
